# Left atrial size and echocardiographic diastolic parameters as predictors of incident atrial fibrillation in older hospitalized patients

**DOI:** 10.1007/s40520-025-02936-6

**Published:** 2025-02-14

**Authors:** Yan Yin, Yanguang Li, Lili Wang, Qiaoyuan Li, Xu Liu, Zhipeng Hu, Jiawei Zhang, Tao Zhang, Zhuo Liang, ShaoMin Chen, Yunlong Wang

**Affiliations:** 1 Department of Cardiology, Beijing Anzhen Hospital, Capital Medical University, Beijing, China; 2 Department of Cardiology and Institute of Vascular Medicine, Peking University Third Hospital, Beijing, China

**Keywords:** Echocardiography, Left atrial size, Older hospitalized population, Atrial fibrillation, Longitudinal study

## Abstract

**Background:**

The associations between left atrial (LA) size, echocardiographic diastolic parameter (E/A ratio), and incident atrial fibrillation (AF) in older inpatients remain underexplored.

**Aims:**

This study aimed to evaluate the relationship between LA size, E/A ratio, and AF risk in older hospitalized patients.

**Methods:**

Between January 2015 and May 2023, a total of 2,615 older inpatients (aged ≥ 65 years) were enrolled in this retrospective longitudinal study. Left atrial diameter (LAD) and E/A ratio were measured using transthoracic echocardiography.

**Results:**

Over a median follow-up of 844 days (IQR: 331–1355 days), 209 patients (8.0%) experienced at least one incident of AF. After adjusting for covariates, large LA and high E/A ratio were significantly associated with incident AF, with an 11% increase in risk for each 1 mm increase in LAD over 35 mm (adjusted HR: 1.11, 95% CI: 1.10–1.13) and a 30% increased risk per standard deviation increase in E/A ratio when E/A ratio exceeded 0.65 (adjusted HR: 1.30, 95% CI: 1.23–1.37), P < 0.001. The influence of LA size and E/A ratio on incident AF was more pronounced in the younger subgroup of older adults. Incorporating LAD and E/A ratios into the CHA2DS2-VASc score improved its predictive accuracy (AUC _increase_ = 0.168, P < 0.001).

**Discussion:**

This study shows that LA size and E/A ratio are key predictors of AF in hospitalized older patients, with age influencing their predictive value. Incorporating these factors into the CHA2DS2-VASc score enhances risk stratification and highlights the need for early AF screening in this group.

**Conclusions:**

In hospitalized older patients, large LA and high E/A ratio are associated with incident AF, and these associations are more pronounced in younger individuals. LAD and E/A ratios provide incremental predictive value for AF beyond the CHA2DS2-VASc score.

**Graphical Abstract:**

LA, left atrium; ASE: American Society of Echocardiography; E, mitral inflow velocity in the early diastolic phase; A, mitral inflow velocity in the late diastolic phase; AF: Atrial Fibrillation.

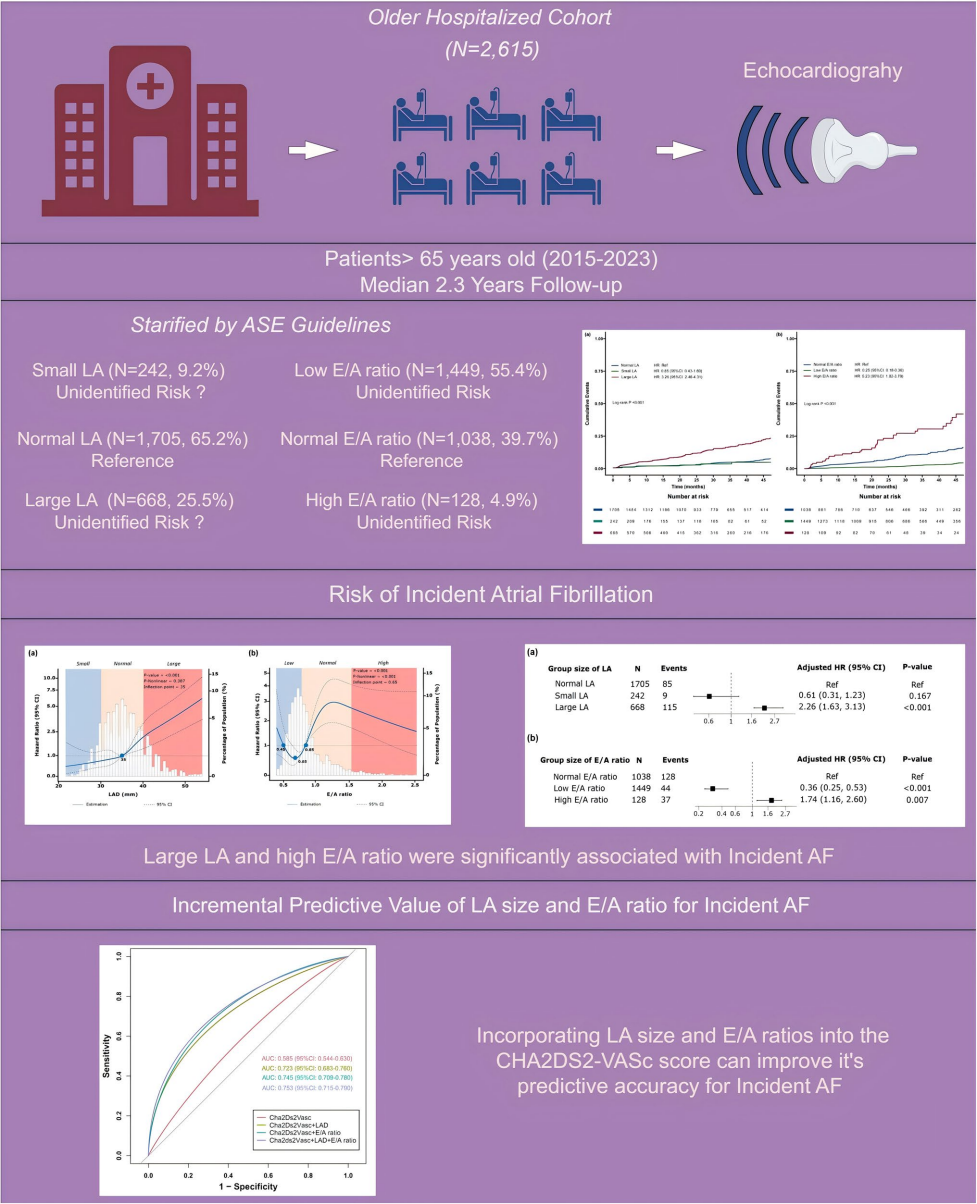

**Supplementary Information:**

The online version contains supplementary material available at 10.1007/s40520-025-02936-6.

## Introduction

Atrial fibrillation (AF) is the most prevalent arrhythmia, affecting an estimated 46.3 million people worldwide [1]. Its incidence increases exponentially after 65 years of age, affecting approximately 7.2% of individuals in this age group [2]. Early detection of AF is crucial for implementing preventive therapies and improving clinical outcomes. However, evidence predicting AF in the aging population remains limited. Owing to its often paroxysmal presentation and subtle symptoms, nearly one million cases of AF remain undiagnosed in the United States alone [3]. Notably, up to 20% of patients with silent AF without an implantable cardiac electronic device experience stroke as the first manifestation of their arrhythmia, underscoring a critical diagnostic gap [4]. Given the high prevalence, associated complications, and poor prognosis of AF in individuals over 65 years, developing robust predictive tools is of significant clinical importance.

Left atrial (LA) enlargement and left ventricular (LV) diastolic dysfunction are well-established markers of structural remodeling in AF and are linked to worse prognosis in patients with AF [5]. Left atrial diameter (LAD) and echocardiographic diastolic parameters, such as the E/A ratio, are commonly used in transthoracic echocardiography to evaluate LA size and LV diastolic function [6]. However, their utility in predicting AF events in older individuals remains unclear. We hypothesized that LAD and E/A ratio could serve as clinically valuable predictors of incident AF in older patients. Additionally, we investigated the interaction of increasing age with LAD and E/A and their combined impact on AF risk. To test this hypothesis, we conducted a longitudinal observational cohort study of older inpatients without AF at admission.

## Methods

### Study design and patients

This retrospective study utilized data from the electronic healthcare database of the Peking University Third Hospital (PUTH), a comprehensive medical institution in Beijing, China, serving over 4 million outpatients and approximately 140,000 inpatients annually [7]. The database contains patient information such as demographics, medical history, diagnostic and treatment records, physical examination results, laboratory findings, imaging data (via the Picture Archiving and Communications System [PACS]), and discharge outcomes. Each patient was assigned a unique registration number for accurate medical record tracking during hospitalization, and diagnoses were coded using the International Classification of Diseases, Ninth (ICD-9) and Tenth (ICD-10) Revisions. The study population included older inpatients (≥ 65 years) discharged between January 1, 2015, and May 1, 2023. Patients with a prior diagnosis of AF identified using ICD-9/ICD-10 codes, self-reported history, or admission electrocardiogram findings were excluded. Additional exclusion criteria included baseline left ventricular ejection fraction < 50%, history of cardiac surgery, congenital heart disease, cardiac device implantation, regional ventricular wall motion abnormalities, cardiomyopathies, connective tissue disorders, and primary valvular diseases, like valvular endocarditis or prolapse.

### Echocardiography

Echocardiograms were obtained by an experienced sonographer and analyzed by blinded echocardiography experts. Triplicate echocardiographic measurements followed the guidelines of the American Society of Echocardiography (ASE) [8] and the European Association of Cardiovascular Imaging (EACI) [9] using the Vivid E9 system (GE Healthcare, Milwaukee, Wisconsin, USA). Left atrial diameter (LAD) was measured in the parasternal long-axis view (2D or M-mode). Peak mitral valve velocities during early (E-wave) and late diastole (A-wave) were obtained from apical four-chamber views. Reference LAD values were categorized as small (≤ 29 mm for males and ≤ 26 mm for females), normal (30–40 mm for males, 27–38 mm for females), and enlarged (≥ 41 mm for males and ≥ 39 mm for females). The E/A ratio was categorized as impaired relaxation (E/A < 0.75), normal (0.75 < E/A < 1.5), and restrictive (E/A > 1.5) based on ASE/EACI criteria [8, 9].

### Outcomes

The primary endpoint was incident AF, defined as an electrocardiogram or 24-h Holter monitoring showing absent repetitive P waves and irregular RR intervals lasting at least 30 s. Data were censored at AF diagnosis or the last follow-up. This study was approved by the Ethics Committee of PUTH in accordance with the Declaration of Helsinki.

### Statistical analysis

Continuous variables were reported as median (IQR) for non-normal distribution or mean ± SD for normal distributions, while categorical variables were presented as percentages. The LAD and E/A ratios were analyzed separately as continuous variables or categorized according to sex-specific ASE/EACI criteria [8, 9]. The Kruskal–Wallis test or Wilcoxon rank sum test was used for continuous variables, and the Chi-square test or Fisher’s exact test was used for categorical variables. Poisson models were used to estimate 95% confidence intervals (CIs) for the incidence of clinical outcomes. Kaplan–Meier curves estimated survival probabilities without primary outcomes across the E/A ratios and LAD categories. Association between LAD, E/A ratio, and outcomes were analyzed using Cox proportional regression analysis, with subjects stratified into young-old group (< 75 years) and middle-to-oldest old group (≥ 75 years), as per World Health Organization (WHO) criteria [10]. The proportional hazard assumption was assessed using the scaled Schoenfeld residual test. Baseline characteristic comparisons utilized only available non-imputed data to ensure the integrity and accuracy of these assessments. Potential non-linear associations among baseline LAD, E/A ratio, and outcomes were explored using restricted cubic splines (RCS) fitted to multivariable Cox models. Knots for the spline models were selected based on the Akaike information criterion minimization. Interaction terms like LAD-age) and E/A ratio-age were included to evaluate the interaction of age with these variables on a continuous scale. Splines were generated using the estimated hazard ratios (HR), and all models satisfied the proportional hazard assumptions. All statistical analyses were performed using SPSS software (version 26.0; SPSS Inc., Chicago, IL, USA) and R Statistics (version 4.5.2; R Foundation for Statistical Computing, Vienna, Austria). The key packages used were survival, *survival*, *survminer*, *rms*, *rmda,* and *pROC*.

## Results

### Baseline characteristics

A total of 2,615 patients were enrolled between January 2015 and May 2023. The median age was 73 years (IQR: 69–79 years), with 45% (N = 1,171) aged 75 years or older and 47.2% (N = 1,233) female. Among the patients, 9.3% (N = 242) had a small left atrium (LA), 65.2% (N = 1705) had a normal-sized LA, and 25.5% (N = 668) had an enlarged LA. Regarding E/A ratio classification, 1,449 patients (55.4%) had a low E/A ratio, 1,038 (39.7%) exhibited a normal E/A ratio, and 128 (4.9%) had a high E/A ratio (Table 1). Compared to participants without AF, those with AF were significantly older (77 vs. 73 years, P < 0.001), had a larger LAD (39 vs. 35 mm, P < 0.001), and exhibited a higher E/A ratio (1.03 vs. 0.71, P < 0.001) (Supplemental Table 1). The incidence of AF was lower in patients with small or normal-sized LA than in those with severe LA enlargement, with a similar trend across E/A ratio categories (P < 0.001). Additionally, significant differences in Left Ventricular End-Diastolic Diameter (LVEDD), Left Ventricular Ejection Fraction (LVEF), Interventricular Septal Thickness (IVST), Left Ventricular Posterior Wall Thickness (LVPWT), Pulmonary Artery Systolic Pressure (PASP), the peak mitral annular systolic velocity (s’), the peak mitral annular early diastolic velocity (e’) and E/e’ ratio were also noted among the groups.

**Table 1:** Patient demographics and baseline characteristics

Characteristic	Small LA, N = 242^1^	Normal LA, N = 1,705^1^	Large LA, N = 668^1^	p-value	Low E/A ratio, N = 1,449^1^	Normal E/A ratio, N = 1,038^1^	High E/A ratio, N = 128^1^	p-value
Age	74 (70, 79)	73 (68, 78)	75 (69, 80)	< 0.001^2^	74 (69, 79)	73 (68, 78)	76 (70, 80)	0.013^2^
Female	138 (57.0%)	844 (49.5%)	251 (37.6%)	< 0.001^3^	671 (46.3%)	498 (48.0%)	64 (50.0%)	0.573^3^
Incident AF	9 (3.7%)	85 (5.0%)	115 (17.2%)	< 0.001^3^	44 (3.0%)	128 (12.3%)	37 (28.9%)	< 0.001^3^
CAD	40 (16.5%)	396 (23.2%)	213 (31.9%)	< 0.001^3^	370 (25.5%)	240 (23.1%)	39 (30.5%)	0.123^3^
COPD	24 (9.9%)	60 (3.5%)	20 (3.0%)	< 0.001^3^	51 (3.5%)	48 (4.6%)	5 (3.9%)	0.380^3^
HTN	97 (40.1%)	818 (48.0%)	342 (51.2%)	0.012^3^	718 (49.6%)	490 (47.2%)	49 (38.3%)	0.039^3^
Diabetes	34 (14.0%)	371 (21.8%)	139 (20.8%)	0.022^3^	323 (22.3%)	206 (19.8%)	15 (11.7%)	0.011^3^
Heart Failure	12 (5.0%)	89 (5.2%)	94 (14.1%)	< 0.001^3^	85 (5.9%)	78 (7.5%)	32 (25.0%)	< 0.001^3^
Thyroid Disease	3 (1.2%)	46 (2.7%)	9 (1.3%)	0.074^3^	30 (2.1%)	27 (2.6%)	1 (0.8%)	0.439^4^
CHA2DS2-VASc	3.00 (2.00, 3.75)	3.00 (2.00, 4.00)	3.00 (2.00, 4.00)	0.002^2^	3.00 (2.00, 4.00)	3.00 (2.00, 4.00)	3.00 (2.00, 4.00)	0.015^2^
LVEDD	43.0 (40.0, 45.8)	46.0 (43.0, 48.7)	50.0 (47.0, 54.0)	< 0.001^2^	46.3 (43.2, 49.3)	47.0 (44.0, 50.0)	47.0 (44.0, 50.0)	< 0.001^2^
LVEF	71 (67, 73)	70 (67, 73)	67 (62, 71)	< 0.001^2^	70 (66, 73)	69 (66, 72)	69 (66, 72)	< 0.001^2^
IVST	8.00 (7.20, 8.84)	8.50 (7.80, 9.20)	9.00 (8.20, 9.90)	< 0.001^2^	8.60 (7.80, 9.40)	8.50 (7.70, 9.23)	8.50 (7.70, 9.23)	< 0.001^2^
LVPWT	7.70 (7.00, 8.38)	8.10 (7.50, 8.90)	8.60 (7.90, 9.40)	< 0.001^2^	8.20 (7.50, 9.00)	8.00 (7.40, 8.90)	8.00 (7.40, 8.90)	< 0.001^2^
MV_E	0.70 (0.56, 0.83)	0.69 (0.59, 0.81)	0.73 (0.60, 0.92)	< 0.001^2^	0.69 (0.58, 0.83)	0.70 (0.59, 0.84)	0.70 (0.59, 0.84)	< 0.001^2^
MV_A	0.94 (0.80, 1.09)	0.95 (0.82, 1.10)	0.94 (0.77, 1.10)	0.458^2^	0.96 (0.83, 1.10)	0.93 (0.79, 1.09)	0.93 (0.79, 1.09)	< 0.001^2^
E/A ratio	0.72 (0.61, 0.83)	0.71 (0.62, 0.82)	0.82 (0.67, 1.14)	< 0.001^2^	0.64 (0.57, 0.69)	0.87 (0.80, 1.03)	0.87 (0.80, 1.03)	< 0.001^2^
PASP	29 (26, 33)	29 (25, 32)	30 (26, 36)	< 0.001^2^	28 (24, 32)	30 (27, 35)	30 (27, 35)	< 0.001^2^
s’	9.20 (8.00, 11.00)	10.00 (8.00, 11.00)	9.00 (7.00, 10.00)	< 0.001^2^	10.00 (8.00, 11.00)	9.00 (8.00, 11.00)	9.00 (8.00, 11.00)	< 0.001^2^
e’	8.90 (7.00, 10.00)	9.00 (7.00, 10.00)	8.00 (7.00, 10.00)	0.002^2^	8.00 (7.00, 10.00)	9.00 (8.00, 10.78)	9.00 (8.00, 10.78)	< 0.001^2^
E/e’	7.5 (6.2, 9.2)	7.9 (6.4, 9.8)	9.3 (7.4, 12.8)	< 0.001^2^	7.5 (6.1, 9.3)	8.8 (7.3, 11.1)	8.8 (7.3, 11.1)	< 0.001^2^

*CAD* Coronary artery disease, *COPD* Chronic pulmonary disease, *LA* left atrium, *LVEF* Left ventricular ejection fraction, *LVEDD* Left ventricular external end-diastolic diameter (LVEDD), *IVST* Interventricular septum thickness, *LVPWT* Left ventricular posterior wall thickness, *PASP* Pulmonary artery systolic pressure, *s’* The peak mitral annular systolic velocity, *e’* The peak mitral annular early diastolic velocity, *E* The mitral inflow velocity in the early diastolic phase, *A* The mitral inflow velocity in late diastolic phase

### Associations between LAD and E/A ratio abnormalities with Incident AF

**Fig. 1:**
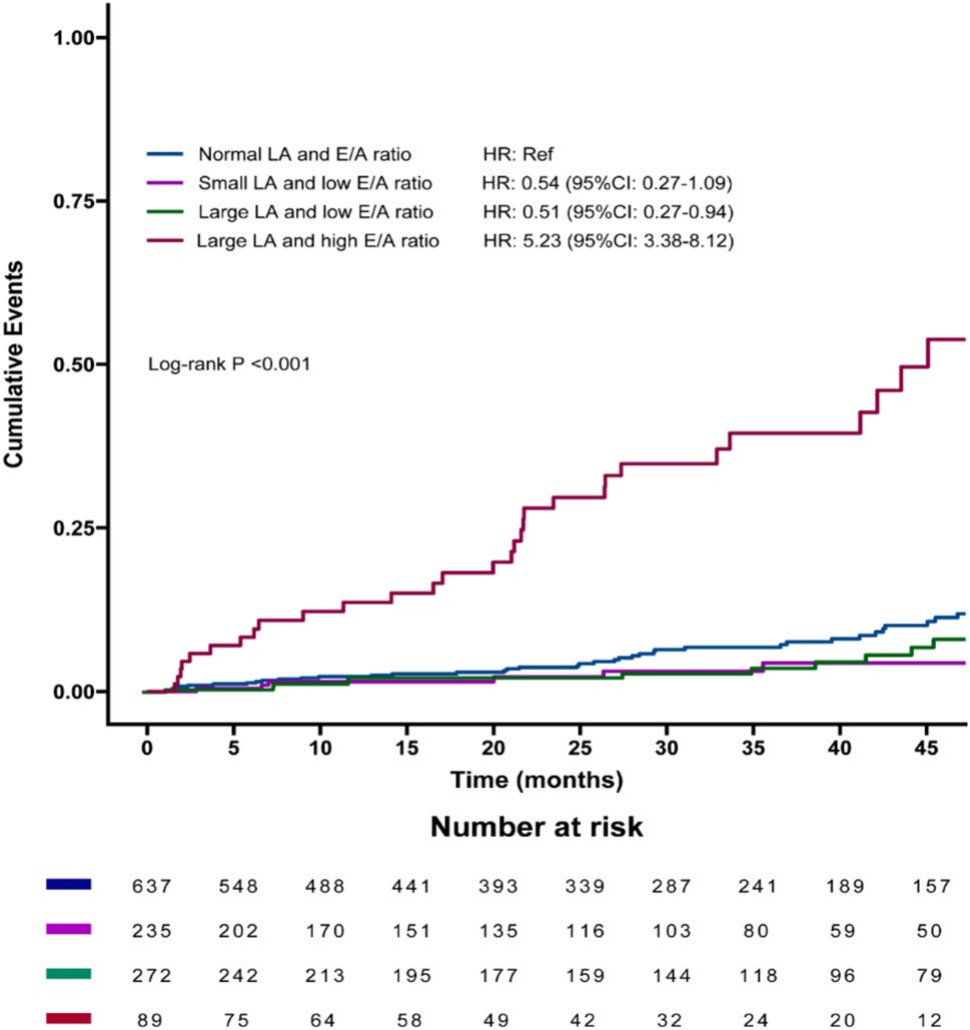
The 45-month cumulative incidence of AF in older inpatients. *AF* Atrial fibrillation

During a median follow-up of 844 days (IQR: 331–1,355 days), 209 (8.0%) patients developed AF (Supplemental eTable1). Patients with small LA and low E/A ratio had the lowest AF events rate (HR: 0.54, 95% CI: 0.27–1.09; P < 0.001), while those with large LA and high E/A ratio had the highest rate of incident AF (HR: 5.23, 95% CI: 3.38–8.12; P < 0.001) (Fig. 1). Significant differences were also observed in LVEDD, LVEF, IVST, LVPWT, PASP, s’, e’, and E/e’ ratio across the groups (Table 1).

**Fig. 2:**
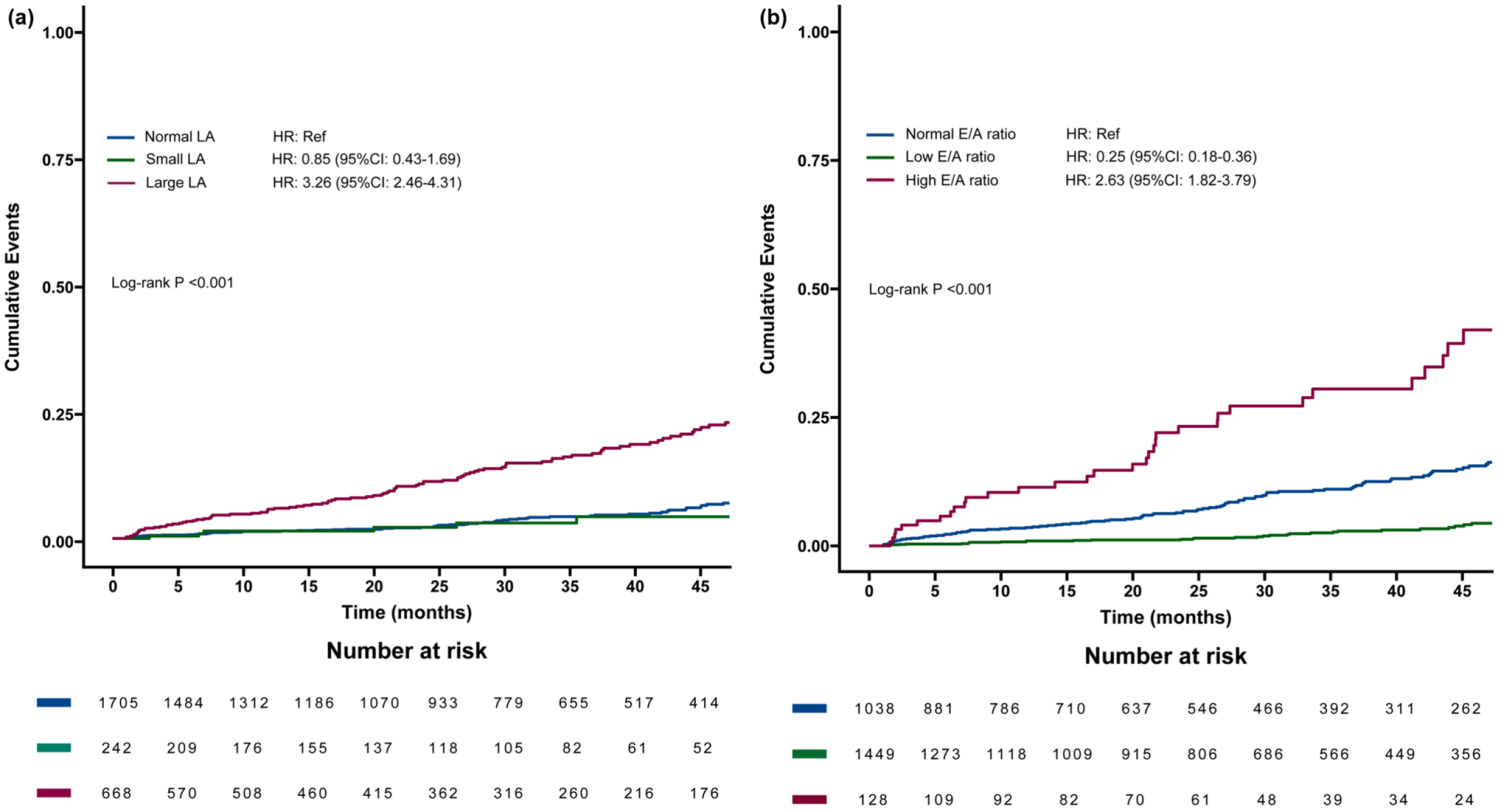
The 45-month cumulative AF incidence in patients categorized by LA size and E/A ratio groups. *LA* Left atrium, *E* Mitral inflow velocity in the early diastolic phase, *A* Atrial inflow velocity in the late diastolic phase

**Fig. 3:**
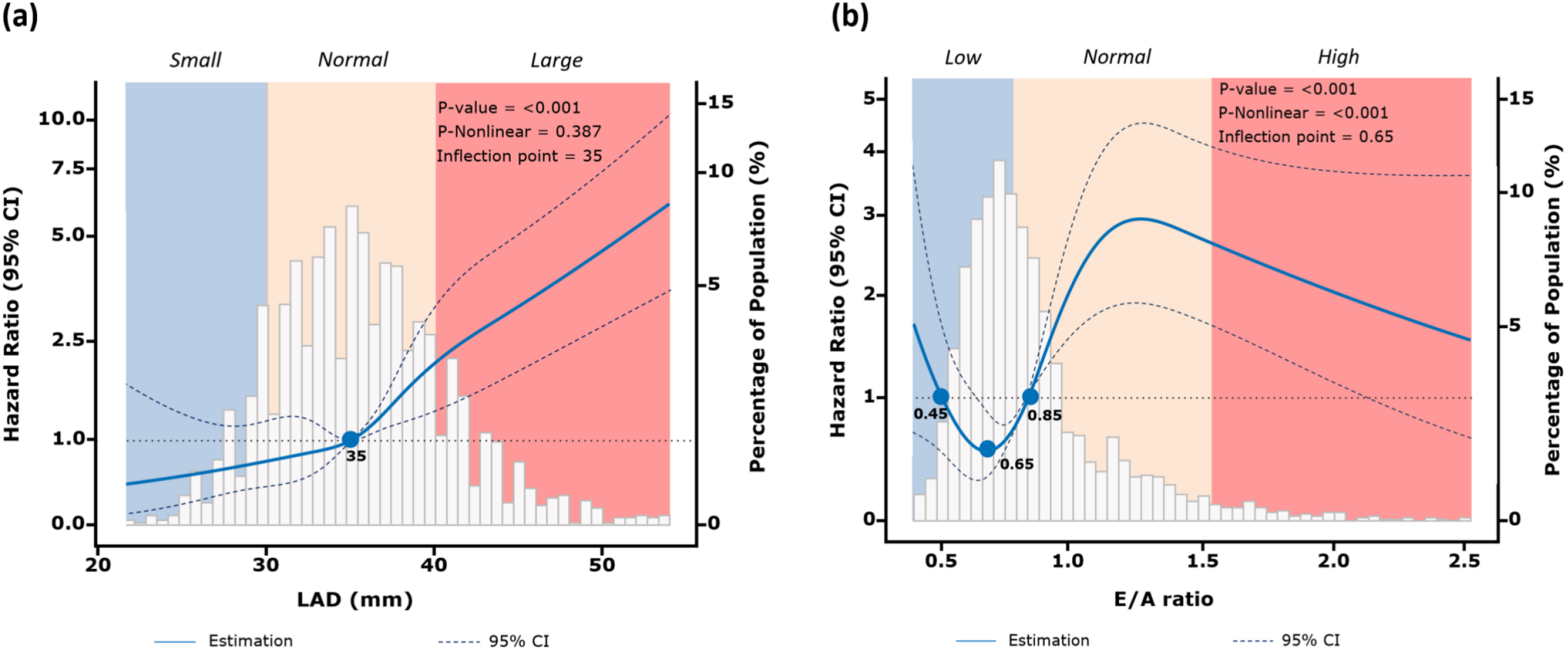
Association between LAD, E/A ratio and AF-free Survival with the RCS function. The cox regression was adjusted for Age, Sex, CAD, COPD, HTN, diabetes and echocardiographic confounders. *LAD* Left atrial diameter, *E* Mitral inflow velocity in the early diastolic phase, *A* Mitral inflow velocity in the late diastolic phase; Atrial fibrillation; *CAD* coronary artery disease, *COPD* Chronic pulmonary disease, *HTN* Hypertension

Compared with the normal LA size group, the large LA group showed a significantly increased risk of AF (HR:3.26, 95% CI: 2.46–4.31; P < 0.001) (Fig. 2a). After adjustment for age, sex, comorbidities, and echocardiographic confounding variables, multivariate Cox regression model showed that patients with enlarged LA had a more than two-fold increased risk of AF compared to those with normal LA size (aHR: 2.26, 95% CI: 1.63–3.13; P < 0.001) (Table 2; Supplemental eFigure1a). In patients with a large LA, the RCS analysis demonstrated a linear association between LAD and incident AF (P < 0.001) (Fig. 3a). For LAD ≥ 35 mm, each 1-mm increase in LAD was associated with an 11% increased risk of incident AF (aHR: 1.11, 95% CI: 1.10–1.13; P < 0.001) (Supplemental eTable2).

**Table 2:** Association between LA size, E/A ratio and arrhythmia-free survival (Cox regression)

Characteristic	Unadjusted					Adjusted		
	**N**	**Event N**	**HR**^**1**^	**95% CI**^**1**^	**p-value**	**aHR**^**1**^	**95% CI**^**1**^	**p-value**
**LA Size**								
Normal LA	1,705	85	Ref	Ref		Ref	Ref	
Small LA	242	9	0.85	0.43,1.69	0.643	0.61	0.31, 1.23	0.167
Large LA	668	115	3.26	2.46, 4.31	< 0.001	2.26	1.63, 3.13	< 0.001
**E/A ratio**								
Normally E/A ratio	1,038	128	Ref	Ref		Ref	Ref	
Low E/A ratio	1,449	44	0.25	0.18, 0.36	< 0.001	0.36	0.25, 0.53	< 0.001
High E/A ratio	128	37	2.63	1.82, 3.79	< 0.001	1.74	1.16, 2.60	0.007

The Cox regression analysis was adjusted for Age, Sex, Coronary artery disease, chronic pulmonary disease, Hypertension, Heart failure, Diabetes, Thyroid disease, and echocardiographic confounding factors

Compared to patients with normal E/A ratio, individuals with low E/A ratio had a significantly lower risk of incident AF (HR: 0.25, 95% CI: 0.18–0.36; P < 0.001), while those with a high E/A ratio had more than double the risk (HR: 2.63, 95% CI: 1.82–3.79; P < 0.001) (Fig. 2b). After adjusting for clinical variables, high E/A ratio remained significantly associated with incident AF (aHR: 1.74, 95% CI: 1.16–2.60; P = 0.007) (Table 2; Supplemental eFigure 1b). A U-shaped relationship was observed between the E/A ratio and AF events, with risk increasing with E/A ratios below 0.65 and above 0.65, with HR < 1 when the E/A ratio ranged between 0.45 and 0.85 (P _overall_ < 0.001, P _non-linear_ < 0.001) (Fig. 3b). With E/A ratio < 0.65, each standard deviation (SD) increase in E/A reduced AF risk by 23% (aHR: 0.73, 95% CI: 0.54–0.99; P = 0.043); conversely, when the E/A ratio was ≥ 0.65, each SD increase in E/A increased AF risk by 30% (aHR: 1.30, 95% CI: 1.23–1.37; P < 0.001) (Supplemental eTable3).

**Fig. 4:**
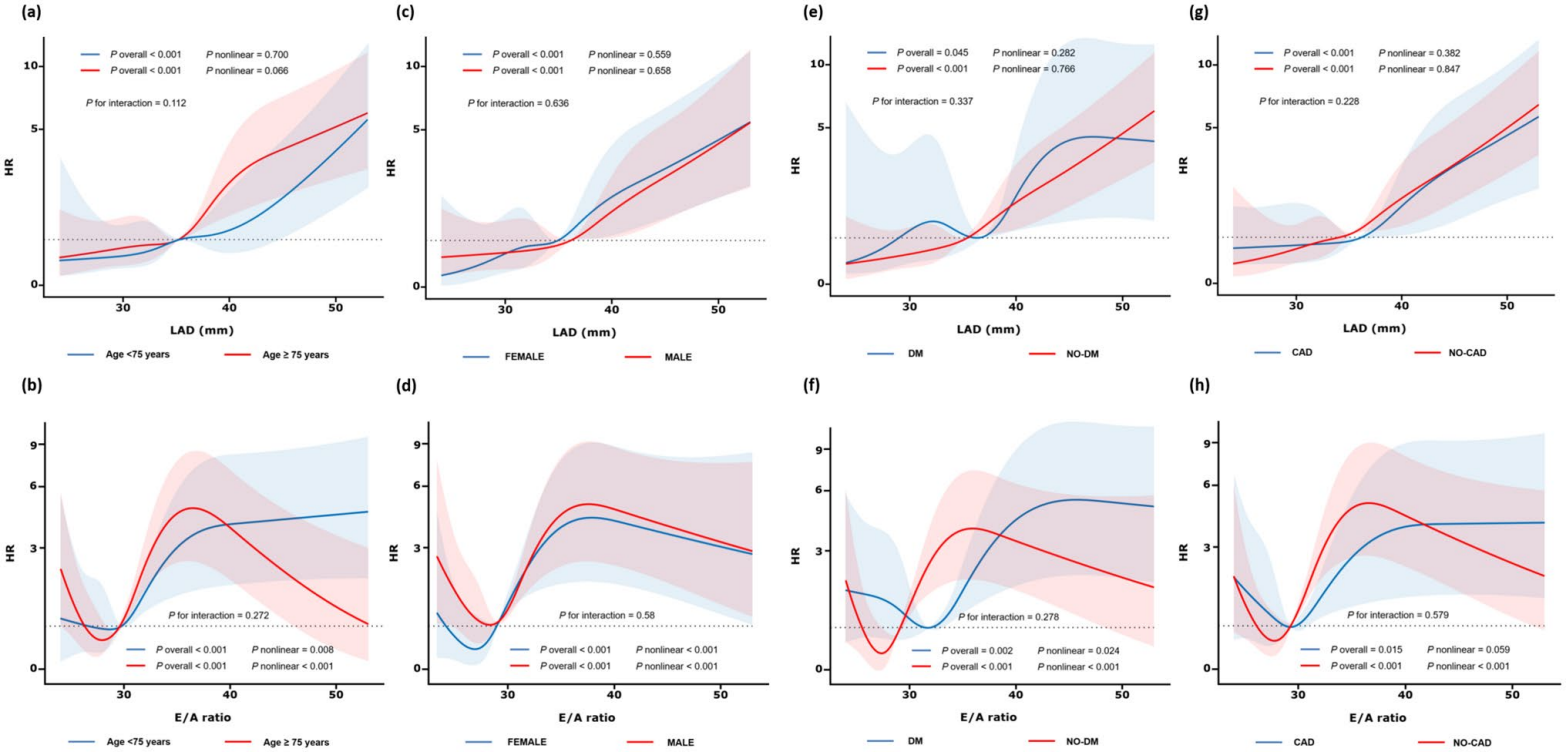
Adjusted Associations of LA size and E/A ratio and incident AF events in Subgroups. *LA* Left atrium, *E* Mitral inflow velocity in the early diastolic phase, *A* Mitral inflow velocity in the late diastolic phase, *AF* Atrial fibrillation

In subgroup analysis by age, sex, diabetes, and coronary disease, linear associations between LAD and AF events and U-shaped associations between E/A ratio and AF events persisted (Fig. 4).

### Subgroup analysis by age

LAD and E/A ratio had a more pronounced effect on incident AF in participants younger than 75 years (Supplemental Fig. 2). The association between LAD and incident AF remained relatively stable across age groups but was slightly stronger in participants younger than 75 (aHR: 1.13 vs. 1.08; 95% CI: 1.07–1.19 vs. 1.05–1.11; P for interaction = 0.112) (Supplemental eFigure 3a). The age-related correlation between E/A ratio and AF risk weakened more significantly with advancing age compared to the LAD*age correlation. Among younger older patients, elevated E/A ratio was significantly associated with a heightened risk of incident AF (aHR: 1.90, 95% CI: 1.35–2.67; P < 0.001), while this increased risk was attenuated in patients above 75 years (aHR: 1.67, 95% CI: 1.03–2.70; P = 0.039) (Supplemental eFigure 3b).

### Improvement in risk prediction with the addition of LA size and E/A ratio

**Fig. 5:**
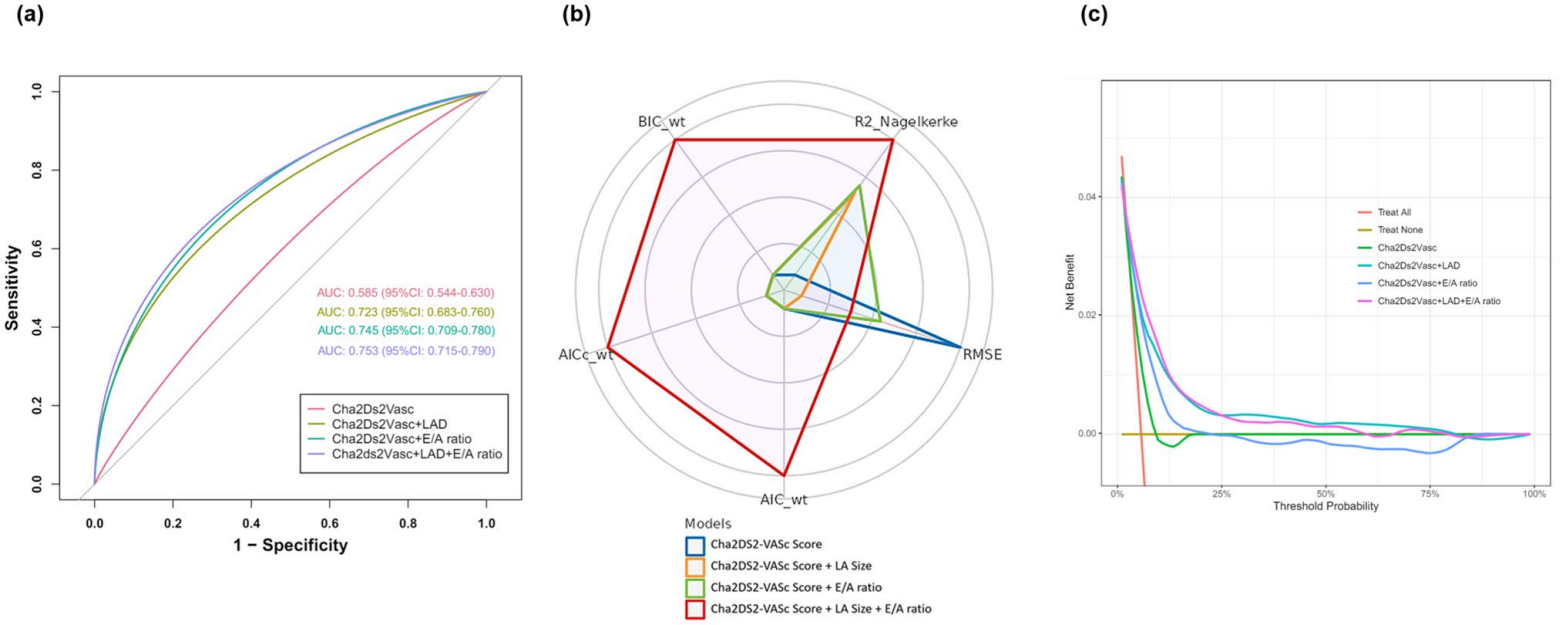
(**a**) ROC curves for CHA2DS2-VASc and CHA2DS2-VASc combined with echocardiographic variables in the prediction of incident AF events. *ROC* Receiver Operating Characteristic curve, *AF* Atrial fibrillation. (**b**) Comparison of Model Performance Indices. *AIC* Akaike information criterion, *BIC* Bayesian information criterion, *RMSE* Root Mean Square Error. (**c**) DCA curves for CHA2DS2-VASc and CHA2DS2-VASc combined with echocardiographic variables. *DCA* Decision Curve Analysis

ROC analysis revealed that the incorporation of echocardiographic variables into the CHA2DS2-VASc score enhanced its predictive performance for incident AF. The combined model of CHA2DS2-VASc with LAD and E/A ratio (AUC = 0.753, 95% CI: 0.715–0.790) demonstrated superior predictive capability compared to the CHA2DS2-VASc score alone (AUC = 0.585, 95% CI: 0.544–0.630) or combined with either LAD (AUC = 0.723, 95% CI: 0.683–0.760) or E/A ratio alone (AUC = 0.745, 95% CI: 0.709–0.780) (P < 0.001, Fig. 5a). The prediction model combining the CHA2DS2-VASc score, LA size, and E/A ratio exhibited strong statistical performance, with a weighted Akaike information criterion (AIC) of 2722.3, Bayesian information criterion (BIC) of 2739.9, high goodness of fit (Nagelkerke’s R^2^ = 0.090), and low prediction error (Root Mean Square Error [RMSE] = 0.291) (Fig. 5b). Decision Curve Analysis (DCA) indicated that the combined model provided greater net benefit and improved clinical applicability (Fig. 5c).

## Discussion

To our knowledge, this is the first study to explore the predictive value of LA size and E/A ratio for incident AF events in older hospitalized patients and to examine age-related trends in these relationships. Findings indicated that an LA diameter < 35 mm and an E/A ratio of 0.45–0.85 significantly reduced AF risk in patients aged > 65 years. The predictive capacity of these echocardiographic parameters varied with age, underscoring the need to identify high-risk older individuals and guide preventive strategies. Previous research [11–13] has linked LA enlargement and left ventricular diastolic dysfunction to AF, with LA size serving as a reliable predictor. This study quantified the echocardiographic risk of AF in individuals over 65 years and demonstrated the enhanced predictive utility of incorporating LA diameter and E/A ratio into the CHA2DS2-VASc score, improving risk stratification for AF prevention among hospitalized aging individuals.

LA size and function are established predictors of AF recurrence after cardioversion or catheter ablation [14–16]. As significant indicators of LA size, the extent of LA remodeling, left ventricular diastolic function, LAD, and E/A ratio reflect structural and functional atrial changes that predispose individuals to arrhythmias. We hypothesized that these changes increase with age, rendering the older population more vulnerable to AF and its complications, such as heart failure and thromboembolic events. In our study, larger LA size and higher E/A ratio were significantly associated with incident AF events, while smaller LA size and lower E/A ratios correlated with lower 5-year AF rates. This suggests that maintaining LAD near the lower normal limit and preserving LA systolic function may reduce AF susceptibility.

Contrary to standard guidelines [8, 9], this study suggests lowering the safe threshold ranges for LA parameters in individuals aged ≥ 65 years due to their heightened susceptibility to atrial and ventricular dysfunction. While LAD exhibited a linear relationship with AF, the E/A ratio demonstrated a U-shaped association, with an optimal range of 0.45–0.85 providing protection against incident AF.

The association between age, the predictive value of LAD, and E/A ratio is biologically plausible. While the predictive value of LAD for AF remained relatively stable in the middle-aged group, the E/A ratio’s predictive value diminished with advancing age, likely due to the progressive prevalence of LV diastolic dysfunction and reduced LA systolic functions in older adults. Such impairments are more pronounced in individuals at high risk of AF than in their younger counterparts, leading to a diminished role of LA systolic function and LV diastolic function in risk assessment.

The CHA2DS2-VASc score is widely used because of its simplicity and accuracy in predicting cerebral infarction risks in patients with AF [17–20]. In a nationwide Danish study [21], the CHA2DS2-VASc score was found to have a C-statistic (similar to the AUC) of 0.606 for predicting stroke in patients with AF, surpassing earlier risk models. A recent cohort study of 22,179 middle-aged adults [22] found that CHA2DS2-VASc ≥ 2 was an independent predictor of incident AF, with an HR of 2.2, and the cumulative incidence of AF increased with CHA2DS2-VASc stratification, with an absolute incidence of > 2% annually whenCHA2DS2-VASc ≥ 4. In the present study, we complemented the CHA2DS2-VASc score by incorporating clinically accessible metrics to substantially improve its predictive ability for incident AF, yielding greater clinical benefit.

In this longitudinal real-world cohort, we identified a proportional relationship between LA size and incident AF events in older individuals alongside a U-shaped relationship between the E/A ratio and new AF events. These findings suggest that the calculated safety thresholds for these parameters were lower than the normal reference ranges recommended by previous guidelines, likely reflecting the effects of atrial aging and ventricular diastolic decompensation in older adults. A key question is whether the LAD and E/A ratio provide greater predictive power for AF risk in individuals younger than 65 years. While our findings imply that early assessment in younger populations may enhance lifetime risk predictions, further research is needed to confirm these associations. Nonetheless, these findings provide a strong rationale for echocardiographic screening in non-cardiac inpatient populations to enable early lifestyle modifications and risk factor management to curb AF progression.

### Limitations

This study has several limitations. Although standard echocardiographic methods were used to assess the LA diameter and E/A ratio, misclassification of LA size may occur in patients with conditions such as patent foramen ovale, atrial septal defect, or atrial septal aneurysm. Additionally, while a small proportion of the study population had a history of heart failure, excluding these patients did not alter the observed associations between echocardiographic variables and prognosis. Future studies with detailed cardiac hemodynamic assessments may further elucidate the mechanisms underlying the poor prognosis in patients with an enlarged LA and high E/A ratios. Moreover, anthropometric measures such as body mass index, body surface area or weight were not recorded in this study, which may limit the consistency of our findings in populations with different body types. To address this concern, we have adjusted for other relevant clinical characteristics and echocardiographic parameters where possible to maximize the stability of our results. Furthermore, because this study was conducted in a Chinese population, the generalizability of the findings to other ethnic groups remains unclear. Lastly, since this study included participants aged 65 to 96 years, our conclusions cannot be extended to adults younger than 65 years or those aged 97 years and older. Broader age-range follow-up studies are needed to explore whether these parameters provide greater predictive value across the full spectrum of adulthood.

## Conclusions

In hospitalized patients aged 65 years and older, a smaller LA and a lower E/A ratio were associated with a reduced risk of incident AF, while a larger LA and a higher E/A ratio correlated with an elevated risk, particularly in young-old individuals.

## Supplementary Information

Below is the link to the electronic supplementary material.

Supplementary file1 (DOCX 489 KB)

## Data Availability

No datasets were generated or analysed during the current study.

## References

[1] Chugh SS, Havmoeller R, Narayanan K et al (2014) Worldwide epidemiology of atrial fibrillation: a Global Burden of Disease 2010 study. Circulation 129:837–847. 10.1161/CIRCULATIONAHA.113.005119 24345399 10.1161/CIRCULATIONAHA.113.005119 PMC4151302

[2] Kornej J, Börschel CS, Benjamin EJ et al (2020) Epidemiology of atrial fibrillation in the 21st century: novel methods and new insights. Circ Res 127:4–20. 10.1161/CIRCRESAHA.120.316340 32716709 10.1161/CIRCRESAHA.120.316340 PMC7577553

[3] Ding EY, Marcus GM, McManus DD (2020) Emerging technologies for identifying atrial fibrillation. Circ Res 127:128–142. 10.1161/CIRCRESAHA.119.316342 32716695 10.1161/CIRCRESAHA.119.316342 PMC8386822

[4] Jaakkola J, Mustonen P, Kiviniemi T et al (2016) Stroke as the first manifestation of atrial fibrillation. PLoS ONE 11:e0168010. 10.1371/journal.pone.0168010 27936187 10.1371/journal.pone.0168010 PMC5148080

[5] Nattel S, Harada M (2014) Atrial remodeling and atrial fibrillation: recent advances and translational perspectives. J Am Coll Cardiol 63:2335–2345. 10.1016/j.jacc.2014.02.555 24613319 10.1016/j.jacc.2014.02.555

[6] Healey JS, Gladstone DJ, Swaminathan B et al (2019) Recurrent stroke with Rivaroxaban compared with Aspirin according to predictors of atrial fibrillation: secondary analysis of the NAVIGATE ESUS randomized clinical trial. JAMA Neurol 76:764–773. 10.1001/jamaneurol.2019.0617 30958508 10.1001/jamaneurol.2019.0617 PMC6583060

[7] Li YG, Bai J, Zhou G et al (2021) Refining age stratum of the C2HEST score for predicting incident atrial fibrillation in a hospital-based Chinese population. Eur J Intern Med 90:37–42. 10.1016/j.ejim.2021.04.014 33975769 10.1016/j.ejim.2021.04.014

[8] Vasconcellos HD, Moreira HT, Ciuffo L et al (2018) Cumulative blood pressure from early adulthood to middle age is associated with left atrial remodelling and subclinical dysfunction assessed by three-dimensional echocardiography: a prospective post hoc analysis from the coronary artery risk development in young adults study. Eur Heart J Cardiovasc Imaging 19:977–984. 10.1093/ehjci/jey086 29982431 10.1093/ehjci/jey086 PMC6102802

[9] Nagueh SF, Smiseth OA, Appleton CP et al (2016) Recommendations for the evaluation of left ventricular diastolic function by echocardiography: an update from the American Society of Echocardiography and the European Association of Cardiovascular Imaging. Eur Heart J Cardiovasc Imaging 17:1321–1360. 10.1093/ehjci/jew082 27422899 10.1093/ehjci/jew082

[10] WHO Definition of an Older or Elderly Person. 2013. Available online: https://www.who.int/healthinfo/survey/ageingdefnolder/en/

[11] Peters DC, Lamy J, Sinusas AJ et al (2021) Left atrial evaluation by cardiovascular magnetic resonance: sensitive and unique biomarkers. Eur Heart J Cardiovasc Imaging 23:14–30. 10.1093/ehjci/jeab221 34718484 10.1093/ehjci/jeab221 PMC8685602

[12] Wang Y, Chao X, Ahmad FD et al (2019) Phoenix dactylifera Protects against Doxorubicin-Induced Cardiotoxicity and Nephrotoxicity. Cardiol Res Pract 2019:1–8. 10.1155/2019/7395239 10.1155/2019/7395239 PMC6942801 31929900

[13] Tsang TSM, Gersh BJ, Appleton CP et al (2002) Left ventricular diastolic dysfunction as a predictor of the first diagnosed nonvalvular atrial fibrillation in 840 elderly men and women. J Am Coll Cardiol 40:1636–1644. 10.1016/s0735-1097(02)02373-2 12427417 10.1016/s0735-1097(02)02373-2

[14] Tian X, Zhang XJ, Yuan YF et al (2020) Morphological and functional parameters of left atrial appendage play a greater role in atrial fibrillation relapse after radiofrequency ablation. Sci Rep 10:8072. 10.1038/s41598-020-65056-3 32415245 10.1038/s41598-020-65056-3 PMC7229104

[15] Moteleb AMAE, Zarif JK, Ali AN (2018) Incidence of atrial fibrosis in non-valvular atrial fibrillation patients and its impact on recurrence after pulmonary vein antral isolation. J Atr Fibrillation 11:1773. 10.4022/jafib.1773 30455829 10.4022/jafib.1773 PMC6207236

[16] Tachmatzidis D, Tsarouchas A, Mouselimis D et al (2022) P-wave beat-to-beat analysis to predict atrial fibrillation recurrence after catheter ablation. Diagnostics (Basel) 12:830. 10.3390/diagnostics12040830 35453877 10.3390/diagnostics12040830 PMC9028701

[17] Dodson JA, Petrone A, Gagnon DR et al (2016) Incidence and determinants of traumatic intracranial bleeding among older veterans receiving warfarin for atrial fibrillation. JAMA Cardiol 1:65–72. 10.1001/jamacardio.2015.0345 27437657 10.1001/jamacardio.2015.0345 PMC5600874

[18] Lowres N, Olivier J, Chao TF et al (2019) Estimated stroke risk, yield, and number needed to screen for atrial fibrillation detected through single time screening: a multicountry patient-level meta-analysis of 141,220 screened individuals. PLOS Med 16:e1002903. 10.1371/journal.pmed.1002903 31553733 10.1371/journal.pmed.1002903 PMC6760766

[19] Lin YS, Chen YL, Chen TH et al (2018) Comparison of clinical outcomes among patients with atrial fibrillation or atrial flutter stratified by CHA2DS2-VASc score. JAMA Netw Open 1:e180941. 10.1001/jamanetworkopen.2018.0941 30646091 10.1001/jamanetworkopen.2018.0941 PMC6324304

[20] Friberg L, Rosenqvist M, Lip GYH (2012) Evaluation of risk stratification schemes for ischemic stroke and bleeding in 182678 patients with atrial fibrillation: the Swedish Atrial Fibrillation cohort study. Eur Heart J 33:1500–1510. 10.1093/eurheartj/ehr488 22246443 10.1093/eurheartj/ehr488

[21] Olesen JB, Lip GYH, Hansen ML et al (2011) Validation of risk stratification schemes for predicting stroke and thromboembolism in patients with atrial fibrillation: nationwide cohort study. BMJ 342:d124. 10.1136/bmj.d124 21282258 10.1136/bmj.d124 PMC3031123

[22] Renda G, Ricci F, Patti G et al (2019) CHA2DS2VASc score and adverse outcomes in middle-aged individuals without atrial fibrillation. Eur J Prev Cardiol 26:1987–1997. 10.1177/2047487319868320 31409109 10.1177/2047487319868320

