# Supplementary material for: Left atrial size and echocardiographic diastolic parameters as predictors of incident atrial fibrillation in older hospitalized patients
Source: Aging Clin Exp Res. 2025 Feb 14;37(1):38. doi: 10.1007/s40520-025-02936-6 (PMC11828783; doi:10.1007/s40520-025-02936-6)

**Supplemental Material**

**Title:** **Left Atrial Size and Echocardiographic Diastolic Parameter and the Risk of New-Onset Atrial Fibrillation in Elderly Hospitalized Population**

| **Supplemental Table 1.** | Baseline Characteristics in patients | Page 2 |
| --- | --- | --- |
| **Supplemental Figure 1.** | Multivariate Cox regression model for associations of LA size, E/A ratio, and incident AF events | Page 3 |
| **Supplemental**  **Table 2.** | Effect of LAD Level on Survival: Adjusted Hazard Ratios from Segmented Cox Regression | Page 4 |
| **Supplemental Table 3.** | Effect of Standardized E/A ratio Level on Survival: Adjusted Hazard Ratios from Segmented Cox Regression | Page 4 |
| **Supplemental Figure 2.** | Hazard Ratio (HR) for incident AF as a Function of Age at Baseline | Page 5 |
| **Supplemental Figure 3.** | Hazard ratio of LAD and E/A ratio for incident AF in age-subgroups | Page 6 |
| **Supplemental Table 4.** | Variance Inflation Factor and Tolerance of variables | Page 7 |
| **Supplemental Figure 4.** | Collinearity of major echocardiographic variables | Page 7 |

**Table1:** Baseline Characteristics in patients

| **Characteristic** | **Incident AF** | | **p-value** |
| --- | --- | --- | --- |
|  | **NO, N = 2,406^1^** | **YES, N = 209^1^** |  |
| **Age** | 73 (69, 78) | 77 (72, 82) | <0.001^2^ |
| **Male** | 1,263 (52.5%) | 119 (56.9%) | 0.217^3^ |
| **LAD** | 35.0 (32.0, 38.0) | 39.0 (35.0, 44.5) | <0.001^2^ |
| **LAS** | 19.0 (17.0, 22.0) | 24.0 (20.0, 29.0) | <0.001^2^ |
| **LVST** | 8.50 (7.80, 9.36) | 8.89 (7.93, 9.60) | 0.009^2^ |
| **LVPWT** | 8.20 (7.50, 9.00) | 8.30 (7.50, 9.00) | 0.303^2^ |
| **LVEF** | 70 (66, 73) | 68 (61, 72) | <0.001^2^ |
| **LVEDD** | 46.6 (43.6, 49.7) | 47.0 (44.0, 51.0) | 0.029^2^ |
| **PASP** | 29 (26, 33) | 34 (28, 41) | <0.001^2^ |
| **s'** | 9.30 (8.00, 11.00) | 8.00 (7.00, 10.00) | <0.001^2^ |
| **e'** | 9.00 (7.00, 10.00) | 9.00 (7.00, 11.00) | 0.203^2^ |
| **E/e' ratio** | 8.1 (6.5, 10.0) | 9.8 (7.3, 13.1) | <0.001^2^ |
| **E/A ratio** | 0.71 (0.62, 0.84) | 1.03 (0.79, 1.29) | <0.001^2^ |
| **Cha2Ds2-Vasc** | 3.00 (2.00, 4.00) | 3.00 (3.00, 4.00) | <0.001^2^ |
| **Coronary artery disease** | 593 (24.6%) | 56 (26.8%) | 0.491^3^ |
| **Chronic pulmonary disease** | 90 (3.7%) | 14 (6.7%) | 0.036^3^ |
| **Hypertension** | 1,149 (47.8%) | 108 (51.7%) | 0.277^3^ |
| **Diabetes** | 510 (21.2%) | 34 (16.3%) | 0.092^3^ |
| **Thyroid Disease** | 56 (2.3%) | 2 (1.0%) | 0.321^4^ |
| ^1^Median (IQR); n (%) |  |  |  |
| ^2^Wilcoxon rank sum test |  |  |  |
| ^3^Pearson's Chi-squared test | | | |
| ^4^Fisher's exact test | | | |

CAD: Coronary artery disease; COPD: Chronic pulmonary disease; HTN: Hypertension; LAD: Left atrial diameter; LAS: Left atrial size; LVEF: Left ventricular ejection fraction; LVEDD: Left ventricular external end-diastolic diameter (LVEDD); IVST: Interventricular septum thickness; LVPWT: Left ventricular posterior wall thickness; PASP: Pulmonary artery systolic pressure; s’: The peak velocity of systolic septal annulus motion; e’: The peak velocity of early diastolic septal annulus motion; E: The mitral inflow velocity in early diastolic phase; A: The mitral inflow velocity in late diastolic phase

Figure 1. Multivariate Cox regression model for associations of LA size, E/A ratio and incident AF events.

LA, left atrium; E, mitral inflow velocity in the early diastolic phase; A, mitral inflow velocity in the late diastolic phase; AF: Atrial Fibrillation

Table 2: Effect of LAD Level on Survival: Adjusted Hazard Ratios from Segmented Cox Regression Analysis

| **Characteristic** | **HR**^1^ | **95% CI**^1^ | **p-value** |
| --- | --- | --- | --- |
| LAD (< 35) | 1.03 | 0.92, 1.15 | 0.62 |
| LAD (≥ 35) | 1.11 | 1.10, 1.13 | <0.001 |
| ^1^HR = Hazard Ratio, CI = Confidence Interval | | | |
| HRs were adjusted for Age, Sex, CAD, COPD, HTN, diabetes, HF, ThroidDisease, EA, IVS, LVPW, LVEDD, PASP, Sm, Em, and LVEF | | | |

Table 3: Effect of Standardized EA Level on Survival: Adjusted Hazard Ratios from Segmented Cox Regression Analysis

| **Characteristic** | **HR per SD**^1^ | **95% CI**^1^ | **p-value** |
| --- | --- | --- | --- |
| EA (< 0.65) | 0.73 | 0.54, 0.99 | 0.043 |
| EA (≥ 0.65) | 1.30 | 1.23, 1.37 | <0.001 |
| ^1^HR = Hazard Ratio, CI = Confidence Interval | | | |
| HRs were adjusted for Age, Sex, CAD, COPD, HTN, diabetes, heart failure, thyroid disease, and other echocardiographic cofounding | | | |

Figure 2: Hazard Ratio (HR) for incident AF as a Function of Age at Baseline

Figure 3: Hazard ratio of LAD and E/A ratio for incident AF in age-subgroups

Table 4: Variance Inflation Factor and Tolerance of echocardiographic indexes

| Variance Inflation Factor and Tolerance | | | | | | | |
| --- | --- | --- | --- | --- | --- | --- | --- |
| Term | VIF | VIF_CI_low | VIF_CI_high | SE_factor | Tolerance | Tolerance_CI_low | Tolerance_CI_high |
| LAS | 1.063826 | 1.022103 | 1.184308 | 1.031420 | 0.9400031 | 0.8443751 | 0.9783748 |
|  |  |  |  |  |  |  |  |
| LVEF | 1.263431 | 1.186029 | 1.373039 | 1.124025 | 0.7914953 | 0.7283112 | 0.8431499 |
| PASP | 1.237208 | 1.163344 | 1.344474 | 1.112299 | 0.8082714 | 0.7437855 | 0.8595910 |
| Sm | 1.750757 | 1.614672 | 1.916970 | 1.323162 | 0.5711814 | 0.5216564 | 0.6193207 |
| Em | 2.174883 | 1.990086 | 2.394172 | 1.474749 | 0.4597948 | 0.4176809 | 0.5024907 |
| EEm | 2.721430 | 2.474389 | 3.009864 | 1.649676 | 0.3674539 | 0.3322409 | 0.4041402 |
| EA | 2.056399 | 1.885152 | 2.260776 | 1.434015 | 0.4862871 | 0.4423260 | 0.5304613 |

Figure 4: Collinearity of major echocardiographic variables


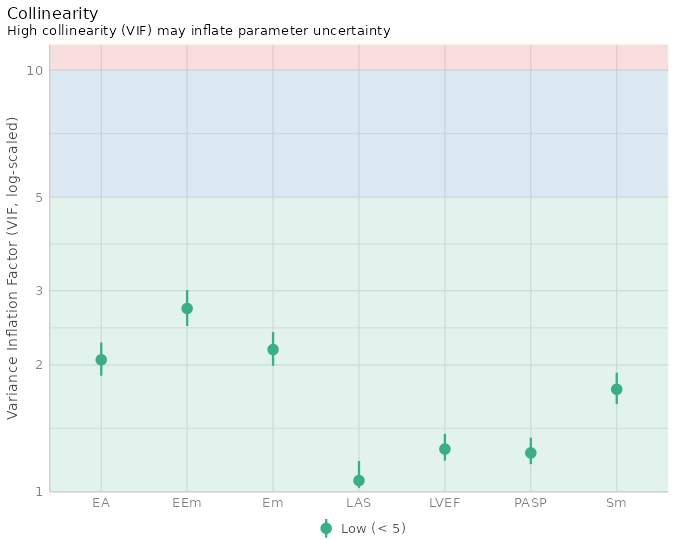

Supplement: Supplementary file 1 — Supplementary file1 (DOCX 489 KB) [file 40520_2025_2936_MOESM1_ESM.docx]
